# Primary adrenal leiomyosarcoma with lymph node metastasis: a case report

**DOI:** 10.1186/s12957-016-0936-z

**Published:** 2016-07-02

**Authors:** Tomoya Onishi, Yutaka Yanagihara, Tadahiko Kikugawa, Noriyoshi Miura, Terutaka Noda, Toshio Kakuda, Riko Kitazawa, Nozomu Tanji

**Affiliations:** Department of Urology, Ehime University Graduate School of Medicine, 454 Shitsukawa, Toon, Ehime 791-0295 Japan; Division of Diagnostic Pathology, Ehime University Graduate School of Medicine, 454 Shitsukawa, Toon, Ehime 791-0295 Japan

**Keywords:** Primary adrenal leiomyosarcoma, IVC reconstruction, Adrenalectomy

## Abstract

**Background:**

Leiomyosarcomas typically originate in smooth muscle cell. Leiomyosarcoma potentially arising from the adrenal gland is an extremely rare mesenchymal tumors associated with delayed diagnosis and poor prognosis.

**Case presentation:**

A 34-year-old man visited our department complaining of right hypochondriac pain. Computed tomography demonstrated a solid mass measuring 5.2 cm in diameter above the right kidney, corresponding to the right adrenal gland, and a lymph node mass, which appeared to have invaded the IVC wall. Right adrenalectomy and lymphadenectomy were performed. A microscopic examination revealed primary adrenal leiomyosarcoma with lymph node metastasis. No adjuvant therapy was performed, and the patient remains recurrence-free at 10 months postoperatively.

**Conclusions:**

We experienced a very rare case of primary adrenal leiomyosarcoma.

Aggressive surgical resection including vascular reconstruction may be associated with improved survival.

## Background

Primary adrenal leiomyosarcoma is exceptionally rare and represents 0.1–0.2 % of intra-abdominal soft tissue malignancies in adults [1]. Its diagnosis is based entirely on the findings of both histological and immunohistochemical evaluations, which are indispensable not only for determining the tumor type but also for predicting the biological behavior. We herein report a rare case of leiomyosarcoma potentially arising from the right adrenal gland in a 34-year-old man who presented with hypochondriac pain.

## Case presentation

A 34-year-old man visited another hospital complaining of right hypochondriac pain. The patient presented with a right adrenal tumor measuring 5.0 cm in diameter and a swollen lymph node measuring 3.5 cm in diameter, which were revealed on plain computed tomography (CT). He was therefore referred to our department for treatment.

**Fig. 1:**
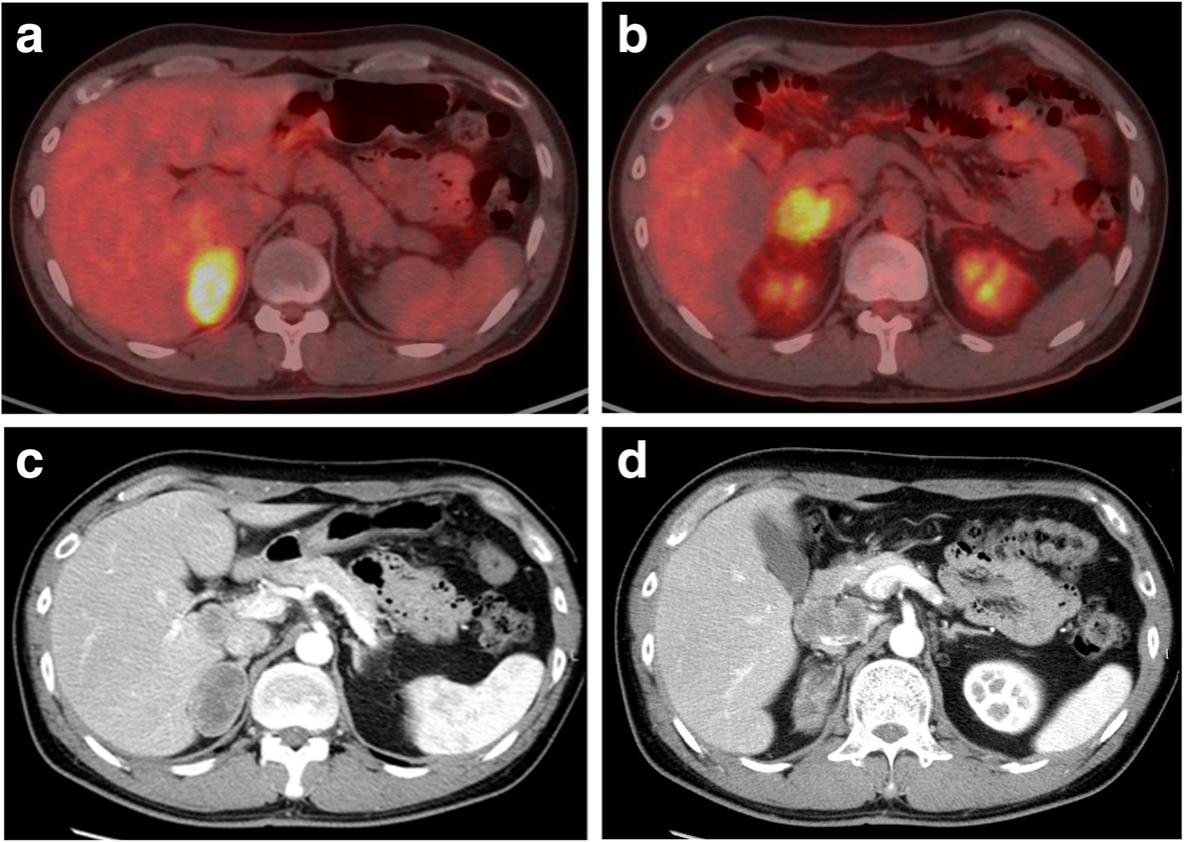
PET/CT FDG uptake was observed in the right adrenal gland (**a**) and lymph node (**b**). A contrast-enhanced CT scan revealed a heterogeneously enhanced solid tumor measuring 5.2 × 3.2 cm in the right adrenal gland (**c**) and a similarly enhanced lymph node mass, which appeared to have invaded the IVC wall, measuring 3.7 cm (**d**)

The patient and his family had been previously healthy. The physical examination on his first presentation was unremarkable. Endocrinological examinations showed normal results. On positron emission tomography/computed tomography (PET/CT), fluorodeoxyglucose (FDG) uptake was observed in the right adrenal gland (SUVmax 7.4) (Fig. 1a) and the lobulated lymph node situated adjacent to the inferior vena cava (IVC) at the level of the proximal portion of the right renal vein (SUVmax 4.5) (Fig. 1b). A contrast-enhanced CT scan revealed a heterogeneously enhanced oval tumor measuring 5.2 × 3.2 cm in the right adrenal gland (Fig. 1c) and a similarly enhanced lymph node mass, which appeared to have invaded the IVC wall, measuring 3.7 × 2.8 cm (Fig. 1d). In addition, the adrenal tumor was densely abutted to the liver and radiologically suspected to infiltrate into it.

**Fig. 2:**
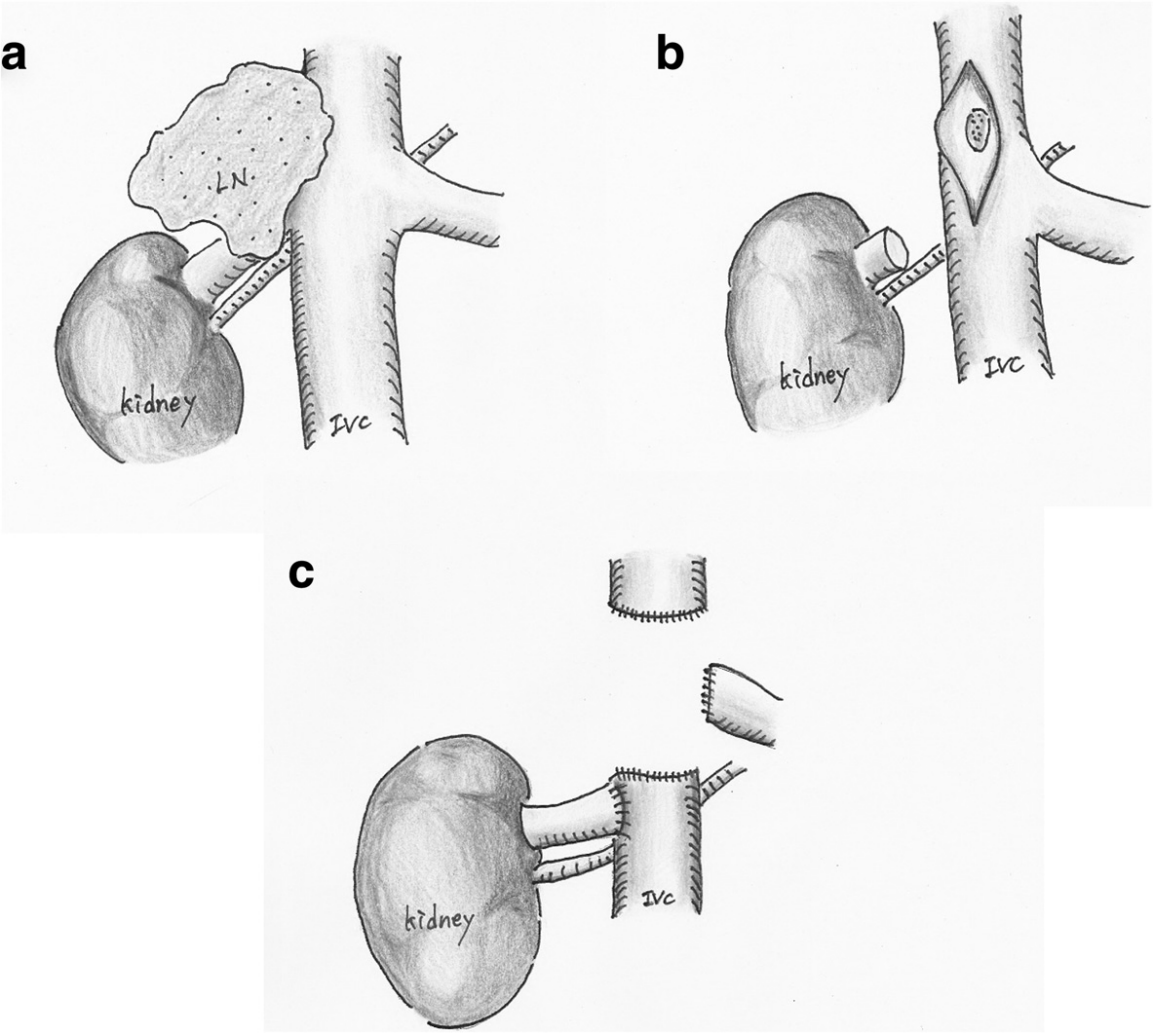
Surgical schematic illustration. **a** A lymph node mass fixed to the IVC. **b** After removal of the tumor, which had invaded the IVC wall, a residual tumor appeared on the opposite site of the IVC wall. **c** The IVC was dissected by Endo GIA™, and the right renal vein was re-anastomosed to the IVC

**Fig. 3:**
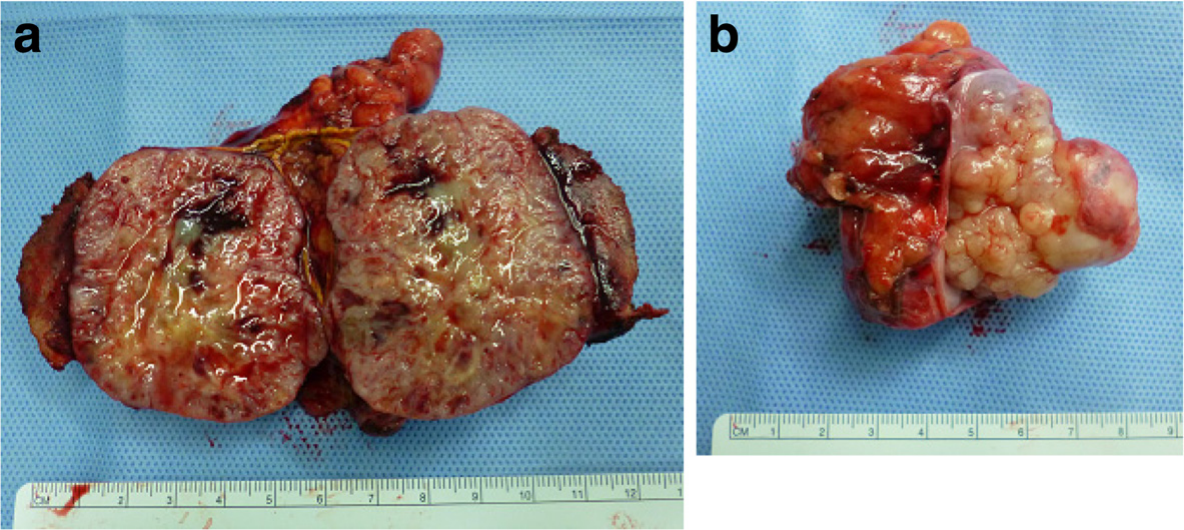
Gross appearance of the tumor sections revealed a grayish-white tumor (**a**). A normal adrenal gland was not identified. The lymph node mass was excised with the IVC wall (**b**)

**Fig. 4:**
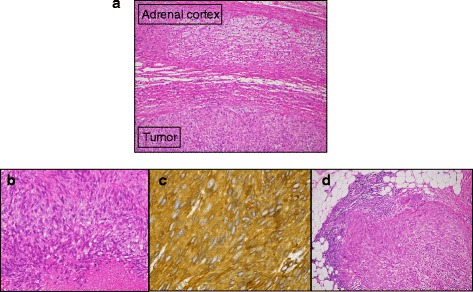
Histological findings. The tumor consisted of spindle-shaped cells with eosinophilic cytoplasm and pleomorphic nuclei (**a**, H&E × 100) (**b**, H&E × 200). The tumor cells were strongly positive for alpha-smooth muscle actin staining (**c**). Lymph node mass (**d**, H&E × 100)

Under a diagnosis of right adrenal cancer and lymph node metastasis, the patient underwent right adrenalectomy and lymphadenectomy in December 2014. First, right adrenalectomy with the preservation of the ipsilateral kidney was performed (Fig. 2a). Because the adrenal tumor adhered to the lower surface of the liver, the tumor was removed in conjunction with a part of the liver, posterior lateral segment (VII). Next, as the lymph node mass fixed to the IVC and could not be separated, it was resected with the invaded IVC wall (Fig. 2b). A residual tumor, however, appeared on the opposite site of the IVC wall. Then, as shown in Fig. 2c, the IVC was dissected by Endo GIA™ (Covidien Japan, Tokyo) and the right renal vein was re-anastomosed to the IVC. The surgically excised adrenal mass displaced a smooth surface, and the cut surface was solid and grayish-white in color with some mucoid areas (Fig. 3a). Small necrotic areas were also present. No normal or residual adrenal gland was macroscopically identified. The gross appearance of the excised lymph node mass demonstrated that the tumor undeniably penetrated the IVC wall (Fig. 3b). Histologically, the normal adrenal gland was displaced by the tumor and presented at the edge of the tumor (Fig. 4a). It consisted of spindle-shaped cells with eosinophilic cytoplasm and pleomorphic nuclei (Fig. 4b). No infiltration to the liver was shown. An immunohistochemical examination revealed that the tumor cells were diffusely positive for alpha-smooth muscle actin (Fig. 4c) and negative for S-100 and CD34. The Ki67 index was approximately 50 % in the hot spot. In the mass excised as a lymph node, the tumor cells existed with the lymphatic tissue (Fig. 4d). Finally, a pathological diagnosis of primary adrenal leiomyosarcoma with lymph node metastasis was made.

Bilateral leg edema was seen right after surgery, which worsened when the patient left his sick-bed. A contrast-enhanced CT scan revealed that there were diffuse thrombi from the IVC to the vein of the lower extremity. Anticoagulant therapy was started to dissolve the thrombi, and the sign gradually improved. The patient remains recurrence-free as of the time of writing.

### Discussion

Leiomyosarcoma arise from various organs containing smooth muscle such as the uterus, alimentary canal, and blood vessels. However, primary adrenal leiomyosarcoma is extremely rare. To the best of our knowledge, only approximately 30 cases have been reported in the English literature [2]. HIV and EBV are suggested to be factors related to its occurrence [1, 2], and it typically presents as a retroperitoneal mass with pain, mass effect, and pressure effect on the surrounding organs. The preoperative diagnosis is difficult. There are no radiologic features available for making the preoperative diagnosis, although imaging studies including CT and MRI are important for assessing the resectability of the tumor and possibility of metastasis. The size or growth rate is also helpful to differentiate malignant tumors from benign ones. In general, adrenal masses larger than 3 cm are suspicious of malignancy [3, 4]. A definitive biomarker for leiomyosarcoma is absent. Lastly, both histological and immunohistological evaluations of the excised tumor are mandatory to make an accurate diagnosis.

It was very difficult to identify the tumor origin in our case, because primary adrenal leiomyosarcoma is extremely rare and lymph node mass penetrated the IVC wall. The origin, leiomyosarcoma, is commonly presumed to be smooth muscle of the blood vessels. However, it was determined by clinical and pathological findings. The tumor which arose from the adrenal gland was bigger than that situated adjacent to IVC. The primary tumor is commonly thought to be bigger than metastatic tumor. In addition, the tumor cells and lymphatic cells were mixed in the mass penetrated the ICV wall. Thus, a final diagnosis of primary adrenal leiomyosarcoma with lymph node metastasis was made in the present case.

Radical surgery is the mainstay of therapy, and the survival depends on tumor size, location, and complete surgical resection with free margins as well as morphologic grading [1]. Despite having slow growth and late metastasis, leiomyosarcomas carry a poor prognosis and unpredictable course with a high incidence of local recurrence [5]. The most important prognostic factor is the ability to achieve a microscopically negative margin. Adjuvant therapy combined with surgery is often used. Strander et al. [6] showed that adjuvant radiotherapy was recommended for the treatment of locally advanced sarcomas. Other factors include the tumor size, location, morphological grading, venous thrombus, and distant metastasis [5, 7, 8]. In our case, dissection of the IVC was required to achieve complete resection. Thus, it was anticipated that the blood flow from the left renal vein bypass gonadal, suprarenal and lumbar veins, and the left renal vein was not reconstructed. Conversely, the right renal vein was re-anastomosed to the IVC. Though leg edema is occasionally problematic after surgery with dissection of the IVC, the development of collateral veins will often resolve it. Our patient also developed leg edema with diffuse thrombi from the IVC to the vein of the lower extremity according to the CT findings. To dissolve the thrombi, anticoagulant therapy was added.

## Conclusions

This is a rare case of primary adrenal leiomyosarcoma with lymph node metastasis. For the time being, early and complete surgical resection is the mainstay management. In our case, dissection of the IVC was required to achieve complete resection. This surgical procedure for dissection of IVC and reconstruction of the right renal vein has been shown to be safe and useful. This case is considered to be useful for future treatment.

## Abbreviation

IVC, inferior vena cava

## References

[1] Nagaraj V, Mustafa M, Amin E, Ali W, Sarsam SN, Darwish A. Primary adrenal leiomyosarcoma in an Arab male: a rare case report with immunohistochemistry study. Case Rep Surg. 2015; doi: 10.1155/2015/702541. 10.1155/2015/702541 PMC4313515 25685588

[2] Zhou Y, Tang Y, Tang J, Deng F, Gong G, Dai Y (2015). Primary adrenal leiomyosarcoma: a case report and review of literature. Int J Clin Exp Pathol.

[3] Singer AA, Obuchowski NA, Einstein DM, Paushter DM (1994). Metastasis or adenoma? Computed tomographic evaluation of the adrenal mass. Clevel Clin J Med.

[4] Yamakita N, Saitoh M, Mercado-Asis LB, Kitada M, Morita H, Yasuda K, Miura K (1990). Asymptomatic adrenal tumor; 386 cases in Japan including our 7 cases. Endocrinol Jpn.

[5] Mencoboni M, Bergaglio M, Truini M, Varaldo M (2008). Primary adrenal leiomyosarcoma: a case report and literature review. Clin Med Oncol.

[6] Strander H, Turesson I, Cavallin-Stahl E (2003). A systematic overview of radiation therapy effects in soft tissue sarcoma. Acta Oncol.

[7] Etten B, van Ijken MGA, Mooi WJ, Oudkerk M, van Geel AN (2001). Primary leiomyosarcoma of the adrenal gland. Sarcoma.

[8] Gulpinar MT, Yildirim A, Gucluer B, Atis RG, Canakci C, Gurbuz C, Caskurlu T. Primary leiomyosarcoma of the adrenal gland: a case report with immunohistochemical study and literature review. Case Rep Urol. 2014; doi: 10.1155/2014/489630. 10.1155/2014/489630 PMC3971559 24716083

